# Quantum analysis of squiggle data

**DOI:** 10.1186/s13040-023-00343-z

**Published:** 2023-10-06

**Authors:** Naya Nagy, Matthew Stuart-Edwards, Marius Nagy, Liam Mitchell, Athanasios Zovoilis

**Affiliations:** 1 College of Computer Science and Information Technology, Department of Networks and Communications, Imam Abdulrahman Bin Faisal University, Dammam, Saudi Arabia; 2 Department of Chemistry and Biochemistry, University of Lethbridge, T1K3M4 Lethbridge, Alberta Canada; 3 Southern Alberta Genome Sciences Centre, University of Lethbridge, T1K3M4 Lethbridge, Alberta Canada; 4 College of Computer Engineering and Science, Prince Mohammad Bin Fahd University, Al Khobar, Saudi Arabia

## Abstract

Squiggle data is the numerical output of DNA and RNA sequencing by the Nanopore next generation sequencing platform. Nanopore sequencing offers expanded applications compared to previous sequencing techniques but produces a large amount of data in the form of current measurements over time. The analysis of these segments of current measurements require more complex and computationally intensive algorithms than previous sequencing technologies. The purpose of this study is to investigate in principle the potential of using quantum computers to speed up Nanopore data analysis. Quantum circuits are designed to extract major features of squiggle current measurements. The circuits are analyzed theoretically in terms of size and performance. Practical experiments on IBM QX show the limitations of the state of the art quantum computer to tackle real life squiggle data problems. Nevertheless, pre-processing of the squiggle data using the inverse wavelet transform, as experimented and analyzed in this paper as well, reduces the dimensionality of the problem in order to fit a reasonable size quantum computer in the hopefully near future.

## Introduction

Novel genomics technologies such as next generation sequencing are revolutionizing the way we generate and use biological data for precision medicine, precision agriculture and other applications. Over the last few years the repertoire of genomic technologies has encompassed novel sequencing platforms, such as the PromethION platform by Oxford Nanopore which enables direct DNA and RNA sequencing for base calling of modified nucleotides. Establishment of the appropriate analysis algorithms and approaches for this data would help to fully realize the potential of this technology.

Squiggle data is the output of nucleotide sequencing by the Nanopore sequencers. Sequencing of just one DNA or RNA sample can produce data in the range of hundreds of megabytes to terabytes. High throughput sequencers such as the Nanopore PromethION perform parallel sequencing of up to 48 samples, resulting in an immense amount of data that requires the development of computationally intensive algorithms for the respective analysis. This amount of data has generated a bottleneck regarding the use of novel genomics technologies at a large scale.

The purpose of this study is to investigate the theoretical advantages quantum methods provide when applied to the analysis of squiggle data. By necessity, the paper also explores the limitations of today’s quantum computers as exemplified by experiments on the IBM QX platform.

The quantum algorithms described in this paper are designed to extract features of the squiggle data. The algorithms exploit quantum parallelism in that arithmetic operations are executed on quantum registers in superposition, or by applying quantum mechanical interference [4].

## Squiggles

Nanopore sequencing is the third generation sequencing technique (TGS) and thus is one of the most recent techniques in practice today [3]. Nanopore sequencing exploits the differences in electrical charge of polynucleotides with different sequences and takes place within a chip that includes a membrane populated with small orifices known as nanopores [5]. A nucleic acids polymer passes from one chamber of the sequencing chip, the *cis* chamber, to the second chamber, the *trans* chamber, through these pores. The strand gets drawn electrophoretically through the pore such that 5 to 12 nucleotides [3] are within the pore at any moment. The presence of nucleotides in the pore affects the current across the membrane between the two chambers and thus gives a reading that depends on the composition of the nucleotides that fill the nanopore at the moment of the reading. Through this process, a sequence of picoampere scale current readings are produced while the polynucleotide is passing through the pore providing the numerical component of the squiggle data. Decoding the squiggle data through a process called basecalling will reconstitute the sequence of nucleotide bases of the original molecule analyzed by the sequencer. The third generation sequencing technique targets a single DNA or RNA molecules for sequencing and owes its success to high speed sequencing of long strands. The maximum length of strands sequenced with TGS varies from 10K bases to millions of bases [8].

The Nanopore squiggle data itself are current readings recorded as integers with range of approximately [300,700], though based on the type of sequencing this range may differ. To represent 400 different values, we need a binary register of nine bits, as $2^9=548 > 400$. As quantum computer memories to date are a mere 5 qubits and up to 15 qubits, computations on 9 bit registers are already unattainable. We envision that a reduction on the value-space will be crucial for quantum applications in any reasonable foreseeable future. Therefore, we investigated ways to reduce the data values with a pre-processing step, while still preserving the features of the squiggle data.

**Fig. 1:**
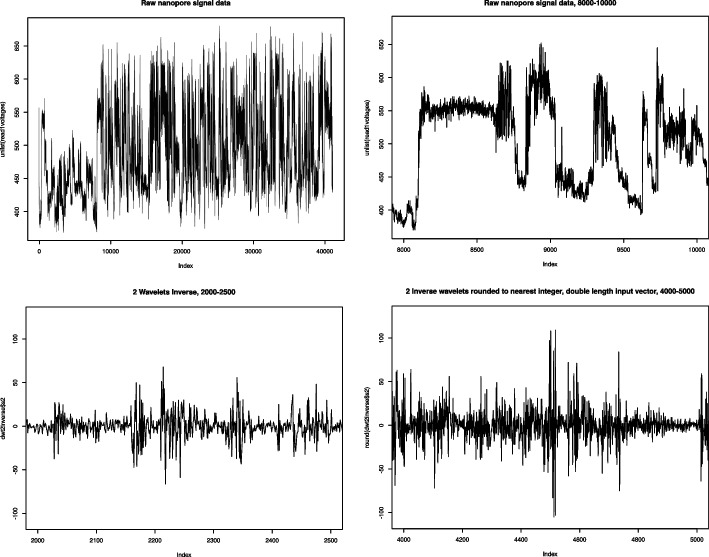
Left-top: Raw signal data from the entire sequence of over 40,000 current measurements. Each index position in x-axis corresponds to one measurement in the squiggle. Right-top: Sub-interval of the raw signal data, from position 8000 to 10000. Left-bottom: The current measurements have been transformed by an *n.levels* = 2 inverse wavelet transformation on positions from 2000 to 2500. Right-bottom: Before applying the inverse wavelet transform, each data point has been recorded twice. The positions are from 4000 to 5000 which directly correlates to the positions in the bottom-left graph

Thus, to reduce the bit-space for processing with near-future quantum computing technology, an inverse discrete wavelet transform was applied to the signal data. Unlike the Fourier transform which reduces the time dimension of the data, the inverse wavelet transform preserves the time dimension and is a reversible operation. As found by Tapinos et al. [14], this transformation applied to Nanopore squiggle data is capable of extracting major features from the data while suppressing noise and analytical performance is preserved. We applied the inverse wavelet transform on direct RNA Nanopore sequencing data of mouse hippocampus brain tissue, using the SQK_RNA002 sequencing kit. The array of current readings has over 40, 000 entries, as shown in Fig. 1, top-left. Implementations were done in R [11], using the waveslim [15] library. In order to see the features, we have worked on a sub-array from positions 8,000 to 10,000, as shown in the figure, top-right. This transformation reduced most values to the range of [-10,10] with large jumps in value in some locations, see Fig. 1, bottom-left. Notice that in this graph, the sub-array points on the x-axis are now from 2,000 to 2,500. By keeping only the values from [-25,25] and recording anything outside of that as a large value, we have reduced the bit-space of the current readings. This way, we have reduced the range values to 50 and the register size is therefore $s=6$, as $2^6=64 > 50$. The analysis of the size of the quantum computer for this bit space can be found in the Discussion and analysis section. Additionally, the variations within the graph can be further enhanced by repeating every value in the original array. This way, the length of the original current array is doubled. In this case, feature variations become stronger, as it can be seen in Fig. 1, bottom-right.

Another reduction operation being attempted is simply rounding the current values to the nearest 5 and using a sliding window of input to normalize the values to a lower bit-space. The sliding window can grow until the values exceed the desired bit-space. This has the advantage of keeping the current values near their original values and applying machine learning techniques similar to previous works in this area. We have not further explored this direction so far.

## Feature definition

The electrical current value of a squiggle measurement is considered to be influenced by approximately 5 bases within the nanopore [12]. Thus, based on one current value only, the type of the base cannot be determined. The sequence of current measurements have to be analyzed together to extract features that are representative for a base or a short sequence of bases. Thus, we looked at features of squiggle data that show modifications in the sequence of current values.

The features of squiggle data that we consider significant for interpretation are

1. values that are close to a constant, showing on the graph as areas with little vertical variation, and
2. values that increase or decrease sharply, showing on the graph as a close to vertical line. These are values that have a large gradient, positive or negative.
3. values that have a peak. These are values that have a large positive gradient on the left and a large (in absolute value) negative gradient on the right.
4. values that have a valley. These are values that have a large (in absolute value) negative gradient on the left and a large positive gradient on the right.

**Fig. 2:**
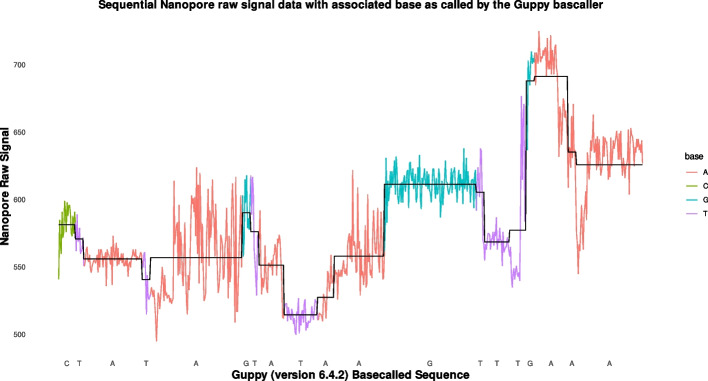
Squiggle features to be detected

Large changes in signal value may indicate a base transition state while inter-base signal variation in the form of peaks, valleys and stalls provide the signal variability required to differentiate base labels. Figure 2 is an excerpt of a graph of squiggle data that shows the features as they appear in reality. The squiggle data was generated by a PromethION sequencer performing direct RNA sequencing with base labelling of signal segments performed by Guppy 6.4.2.

## Quantum bits and operations

The advantage of using quantum operations in the detection of features stems from the added possibilities of quantum states versus classical states together with the specific quantum gates versus classical computations on bits. In particular, properties such as superposition and quantum parallelism can capture multiple operations into one.

### Qubits and quantum gates

The primary quantum feature that is used in our algorithm is superposition, the capability of a collection of qubits to hold multiple superimposed states at the same time. In order to connect the particular nonce value with the value of the Hash, we also need the qubit system to be entangled.

#### Qubits

A general qubit [10] is described in Dirac’s notation as a superposition of the base vector $|0 \rangle$ and $|1 \rangle$.

$$\begin{aligned} q = \alpha |0 \rangle + \beta |1 \rangle , \end{aligned}$$

where the coefficients $\alpha , \beta$, the amplitudes, are complex numbers and the vector is of unitary norm, i.e. $\sqrt{| \alpha |^2 + | \beta |^2} = 1$. For an ensemble of qubits, the formula can be extended naturally. For two qubits, we have

1 $$\begin{aligned} q_{AB} = \alpha |00 \rangle + \beta |01 \rangle + \gamma |10 \rangle + \delta |11 \rangle \end{aligned}$$

with the unitary condition $\sqrt{|\alpha |^2 + |\beta |^2 + |\gamma |^2 + |\delta |^2} = 1$ where $\otimes$ is the tensor product operator.

Generalizing the above, we get that a register, an ensemble of *n* qubits can hold up to $N=2^n$ different values, with various amplitudes. Note that some of the amplitudes may be zero, and the corresponding states can be omitted from the description. In the case when all the coefficients except a single one are zero, the quantum register has a unique value stored in it and is equivalent to a classical register. At the other end of the spectrum, when all coefficients are equal, the quantum register is said to be in a balanced superposition of all possible states. For a register of *n* qubits, the balanced superposition is

2 $$\begin{aligned} q_1 \ldots q_n = \frac{1}{\sqrt{N}} \left( |00...0 \rangle + |00 ... 01 \rangle + ... + |11 ... 1\rangle \right) . \end{aligned}$$

Note that we have omitted the $\otimes$ symbol in the equation above.

#### Measurements

Superposition provides a natural way to apply a function to various inputs (components) in parallel. There is one limitation: we cannot see the work in action. The drawback comes from not being able to extract all the information in a quantum register. The complex coefficients of the components of the superposition represent the probability of each component. For example, if the coefficient of a component is $\alpha$, then the probability of observing that component is $|\alpha |^2$.

When a quantum register is measured, the result is a classical register value and is the value of only one component. The superposition is said to collapse to the state represented by the value. The superposition can collapse to any one of its components, and it obeys the probabilities given by its coefficients. Thus a component with a large coefficient is more likely to be observed than a component with a small coefficient. In the case of a balanced superposition, all the components are equally likely to be observed as a result of a measurement.

It is also impossible to make a copy of a qubit in an unknown state. This property is called the *nonclonability* of qubits and follows from the linearity of the operator.

#### Quantum gates

All regular binary gates, such as NOT, OR, AND, XOR, and similar other gates, exist readily for quantum registers as well. Also, swap operations on the bits left, and right shift operations on registers can be implemented on quantum registers as well.

There are additional gates needed to specify our algorithm in the next section; they are quantum specific. These are the Hadamard gate and the controlled-NOT (CNOT) gate. The NOT gate can also have two controls in which case it is a CCNOT gate. These gates and their generalizations are used to implement Grover’s algorithm for unstructured search, presented in the next subsection. For a general description of quantum gates see [10].

To specify our algorithm, we need the Hadamard gate (*H*) which is applied on a base state $|0 \rangle$ or $|1 \rangle$ to obtain a balanced superposition. When the Hadamard gate is applied to a balanced superposition directly, the state returns to a base state. The Hadamard gate is its own inverse, and therefore, applied twice to a qubit, restores its original state. If a qubit is in a simple state $q_{zero} = |0 \rangle$, then the Hadamard gate transforms $q_{zero}$ into a balanced superposition: $H(q_{zero}) = H(|0 \rangle ) = {\frac{1}{\sqrt{2}}}(|0 \rangle + |1 \rangle ),$ and $H({\frac{1}{\sqrt{2}}}(|0 \rangle + |1 \rangle )) = |0 \rangle = q_{zero}$. For the state $q_{one} = |1 \rangle$, a similar transformations exist: $H(q_{one}) = H(|1 \rangle ) = {\frac{1}{\sqrt{2}}}(|0 \rangle - |1 \rangle )$, with the inverse transformation $H({\frac{1}{\sqrt{2}}}(|0 \rangle - |1 \rangle )) = |1 \rangle = q_{one}$.

The CNOT and the CCNOT gates have two and three inputs respectively and the same number of outputs. There is one data input to which the *not* applies, the other (one or two) inputs are the control qubits. When all control qubits are equal to one, the value on the data input is flipped; otherwise, the data input is left unchanged.

Quantum gates can have an arbitrary number of inputs. A requirement on quantum gates is that the number of inputs is equal to the number of outputs. This is because quantum gates are reversible. They are linear transformations and can be used both from inputs towards outputs as well as from outputs towards inputs. Quantum gates (linear transformations) are information preserving.

### Grover’s algorithm

Grover’s algorithm [6] performs an unstructured search. It was initially developed by Lov Grover to find a specific record, if one exists, in a database of unordered records. Grover’s algorithm has found numerous applications, as it is formulated for a general data format.

Suppose there is a set of data items. This set can be an arbitrary data structure of unordered items or records. The algorithm searches for a record that meets specific criteria. The criteria are formulated as a boolean function, taking in the item and computing a true or false value. We are interested in finding one record on which the boolean function is true.

The algorithm starts with a balanced superposition of all states. This allows us to operate on all the records, in parallel. Then, iteratively, the coefficient (amplitude) that corresponds to solutions states is increased to the disadvantage of the non-solution components in the superposition. After several iterations, the probability of measuring a solution is considerably higher than the probability to measure a non-solution. The failure rate can be as low as $2^{-n}$, where *n*, in this case, is the size of the solution space. For a detailed description of Grover’s algorithm together with a modest size implementation on IBM Quantum QX, see [9].

## Quantum circuits to compute squiggle properties

We hereby show theoretical circuits for the squiggle features defined in the previous section. All these circuits are realizable in theory, though they do need larger quantum memories than available at present.

There are a few constants that apply to all circuits and properties that we consider.

The current values are always within the same range. As discussed before, the range can be taken from raw data or it can be pre-processed. For the analysis of our algorithms, we will use the generic parameter *s* to refer to the size of the input squiggle electrical current value.

In all property calculations, we consider a number of consecutive values that define the feature. the choice on the number of values to be considered may lie with the experimenter. We will denote this number with *n*.

We are ready to see the quantum circuits themselves.

### Quantum circuit for near to constant values

The feature to be determined here is described by a data point $P_0$ that is very close in value to its neighbors. The neighbors, $P_1, P_2, ..., P_{n-1}$ may be predecessors, successors, or half before and half after the main point *P*. Thus, the set *S* of neighbors are within a small range of $P_0$ if the pairwise difference is a small positive or a small negative. This means that $|P_0-P_i| < \epsilon$ for all $i=1, 2, ..., n-1$. The parameter $\epsilon$ depends on the finesse of the definition of *near to constant* data.

**Fig. 3:**
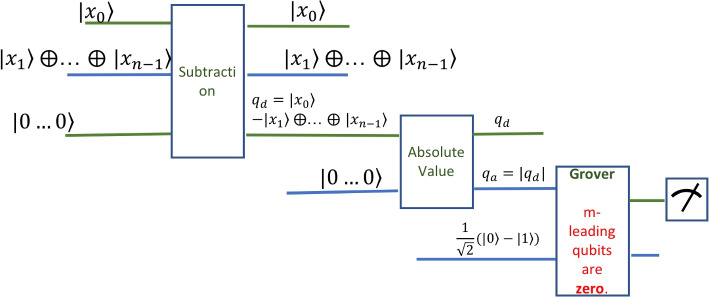
Circuit to determine whether a set of consecutive values are near to constant

The idea of the circuit in Fig. 3, is to have two inputs: first, the value $P_0$ to be compared to, and second, the set *S* of the neighboring values in superposition. Additionally, the circuit has to input two registers of $|0\rangle$ to hold partial and final results, and the extra input to Grover’s circuit. The additional inputs also ensure the circuit is reversible.

The circuit has three stages: the first stage computes the subtraction of two qubit registers, the second stage computes the absolute value of a qubit register, and the last stage is the application of Grover’s algorithm. After stage two, before Grover, all differences are computed in absolute value and they coexist in a superposition of $n-1$ components. A positive result means that all components have a number of leading zeroes, defined by $\epsilon$. Thus, Grover’s algorithm has to enhance the probability to read the non-zero component, if it exists. Thus, if the measurement at the end has only leading zeroes, then then sequence of current values is near-to constant. Otherwise, the test failed.

To analyze this circuit, we will use the breadth and depth measurements. Depth is the size of the qubit registers used and depth is the number of gates along the longest line of the circuit [2]. The breadth of the Near-To-Constant circuit is $4*n+1=O(n)$. The depth of the circuit is given by the depth of the three stages. We ignore the preparation of the superposition input. The first two stages can be implemented in a depth of circuit linear in the size of the input *O*(*n*). The depth of Grover’s algorithm [16] depends on the number of options in the search space, which is $n-1$ in this case. Thus, the depth of Grover’s algorithm is $O(\sqrt{\log {(n-1)}})=O(\log n)$. And the overall depth is the addition of the two, namely *O*(*n*).

### Quantum circuit for sharp increase or decrease of values

The next quantum circuit we developed is targeted to recognize a steady, sharp increase or decrease in the values. As the decrease property is the direct opposite of the increase property, this section describes the circuit for the increase of value in detail and then mentions briefly the differences in the case of decreasing values.

**Fig. 4:**
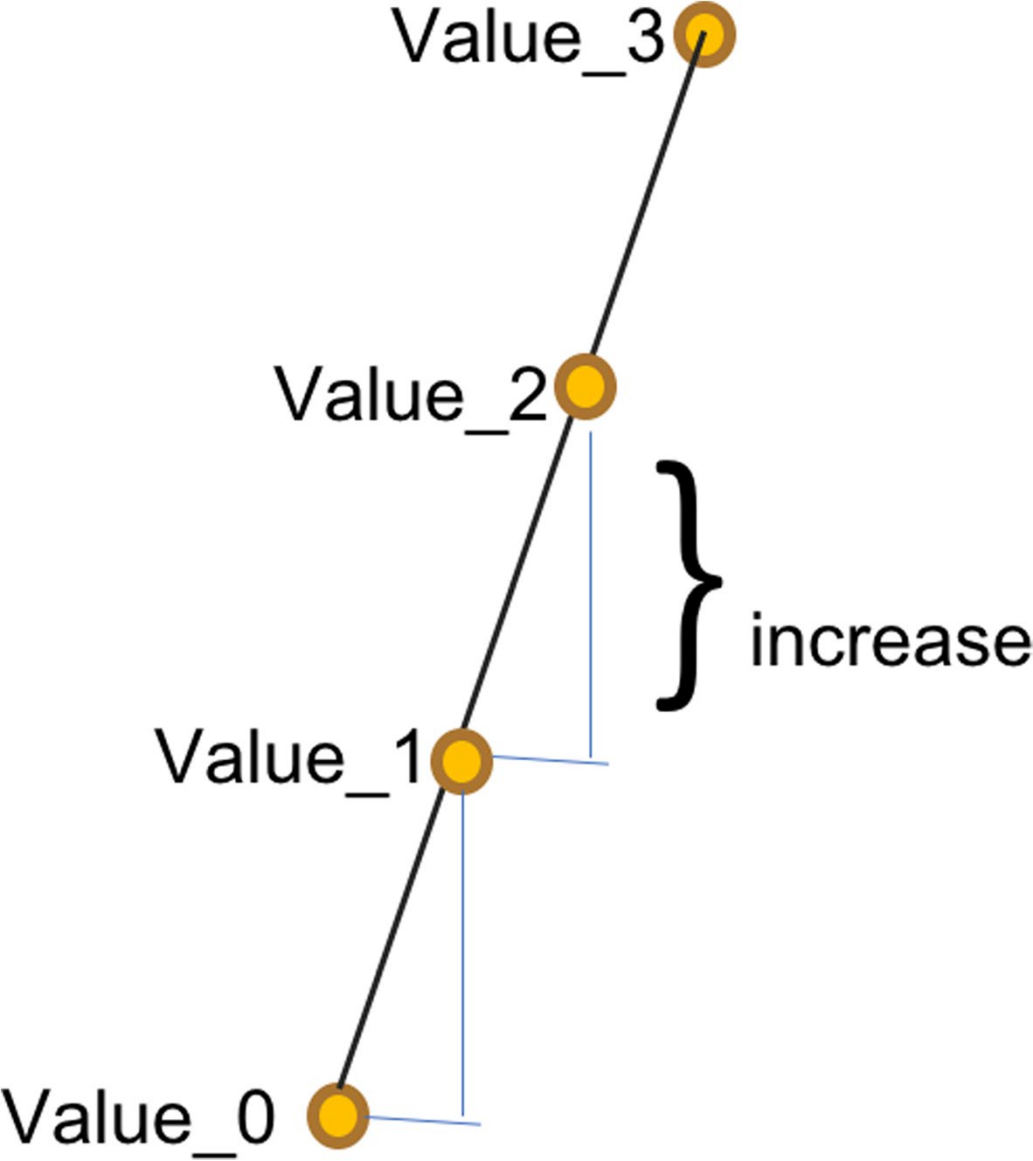
Squiggle values with a sharp increase. For this example, the number of consecutive values to be considered for a sharp increase is $n=4$. The value of the increase considered significant, *incr*, is also shown on the figure

A few constant parameters need to be decided upon at the start. The number of consecutive values that determine a sharp increase is one such parameter. Next, it needs to be clear what a sharp increase means after all, namely the difference (or tangent) that is considered significant. Denote the number of consecutive values by *n*, this resembles the meaning in the previous circuit. The lowest significant increase is denoted by *incr*, which is a threshold value. Figure 4 shows these parameters as they refer to measured squiggle values. Also, we will consider that a current value is stored in a register of size *s*.

Consider an array of *n* consecutive current values for consecutive points $value_i$, where $0 \le i \le n-1$. Thus, in order to detect a sharp increase, we need to compute all partial differences $value_{i+1}-value_i$, for $0 \le i \le n-2$, and compare the difference with the threshold value *incr*. If all $value_{i+1}-value_i \ge incr$ then this array is considered to be a sharp increase. The advantage of using the parallel computer is that we can compute $n-1$ all differences in superposition and then compare the resulting superposition difference to the threshold *incr*.

**Fig. 5:**
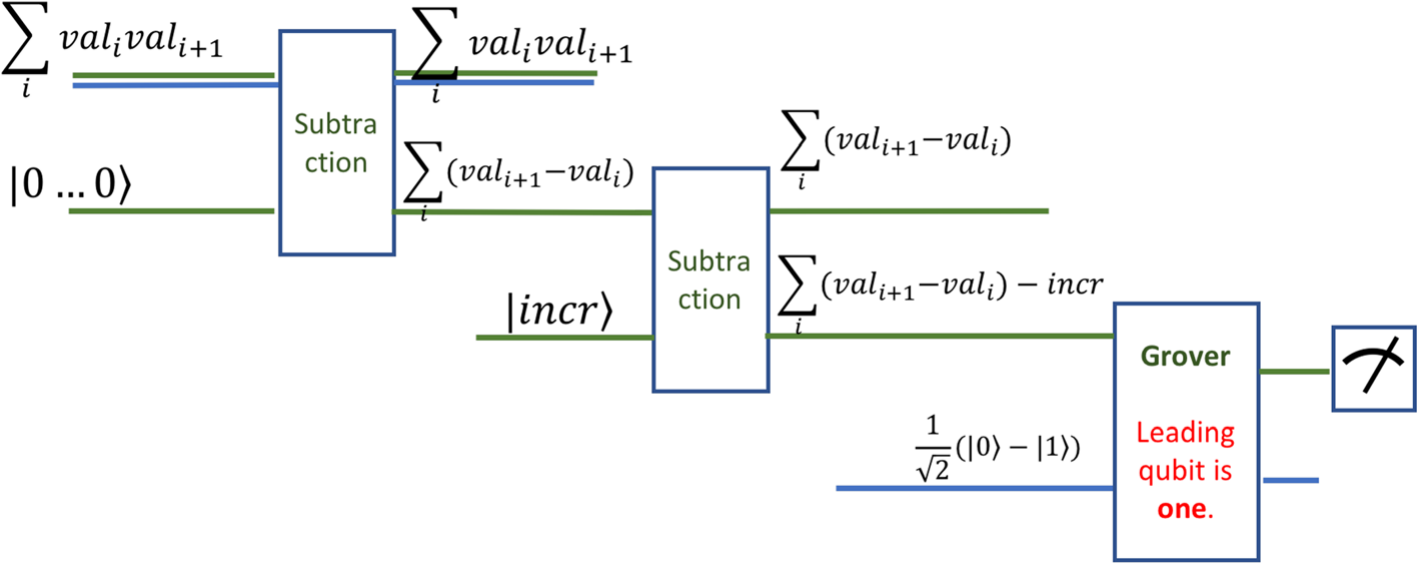
The circuit that detects a sharp increase

The quantum circuit that computes the differences and compares to the threshold is shown in Fig. 5. The structure of the circuit is given by three main stages. The first stage computes the pairwise differences. The second stage compares these differences with the threshold value *incr*. The last stage is the application of Grover’s algorithm to check whether all comparisons were successful.

The data input of the circuit consists of a 2*s* input register. It holds each pair

$$\begin{aligned} (value_{i+1},value_i) \end{aligned}$$

in superposition with all other pairs. Thus, for *n* consecutive values, we have a superposition of $n-1$ terms. This register is described by the state $\sum _{i=0}^{n-1} |value_{i+1} \rangle |value_{i}\rangle$. Additionally, the circuit has inputs to hold the results. The circuits need three inputs for the results, reflecting the three stages. First, the circuit needs a register of size *s* to hold the result of the subtraction. This is set to zero. Second, the comparison circuit needs the threshold value, which can also be considered to be a register of size *s*. Grover’s algorithm needs one qubit input to give the result. This qubit is set as always to a balanced superposition of zero and one, namely $\frac{1}{\sqrt{2}}(|0\rangle -|1\rangle ) = H Not |0\rangle$, obtained after applying a NOT and a Hadamard gate on the base state $|0\rangle$. Formally, the state of the input register is

$$\begin{aligned} S_0 = \sum _{i=0}^{n-1} |value_{i+1} \rangle |value_{i} \rangle \otimes |00...0\rangle \otimes \frac{1}{\sqrt{2}}(|0\rangle -|1\rangle ) \end{aligned}$$

The first stage of the circuit computes the subtraction of all pairs $(value_{i+1},value_i)$. The subtraction is happening on a 2*s* qubit register in superposition. The depth of the circuit is *O*(*s*) which is equivalent to the classical depth of such a circuit. The advantage of the quantum approach is that all subtractions are happening in parallel. Thus, for the classical case, the $s-1$ subtractions are executed in $O(n \times s)$, whereas the quantum version is executed in *O*(*s*) times only. A practical implementation of a subtraction on one qubit only will be shown in the One qubit subtraction circuit section with the gate analysis and accuracy measurement. We may consider that the error of one qubit subtraction accumulates over the length of the register by a factor of 2*s*.

The second stage of the circuit tests whether the result of all subtractions is sharp enough. By sharp enough, we mean that the result of the subtraction is larger than the expected increase. This is another subtraction circuit, where the value *incr* is subtracted from all previous results. If the increase is not sharp enough, this result will be negative. Or, if some increases in the superposition are not sharp enough then the result will have some negative components. This time the subtraction is on registers of size *s*. Thus, the depth of the circuit is again *O*(*s*).

The third stage of the circuit, which is Grover’s algorithm, determines if there are subtractions with a negative result. This translates into testing whether the resulting superposition has a solid leading one, which is determined when all terms of the superposition have a leading one. Thus, Grover’s algorithm increases the probability of one for this one qubit. Note that in this case Grover’s algorithm may be applied on one qubit only. Nevertheless, if $s-1$, the number of terms in the superposition is large, and there is only one negative number in the superposition, then the initial probability of that one is very small and Grover’s algorithm is indeed necessary.

Overall, the circuit to check for a sharp increase has a breadth of *O*(*s*) and a depth of *O*(*s*), which shows a volume of $O(n^2)$. Naturally, for a sharp decrease the circuit needs to reverse the sign of the difference to $| incr \rangle$, and, in the case of success, expect positive results only.

It should be noted that the circuit above detects steady increases in values. The nature of the circuit is that it checks that the threshold is always met by any two consecutive values. Therefore, if only one pair of values do not meet the threshold, the entire increase is rejected. If the rejection rate is too high, adjustments have to be made by the value of the threshold itself *incr*, or by the value of *n*.

### Quantum circuit for peak values and valley values

For the sake of completion, circuits detecting peak and valley values are discussed here, though the circuit is simple. In both cases, we need two circuits described in the previous section, to detect both a sharp increase as well as a sharp decrease. The two circuits need to take into consideration half of the qubits *n*/2 for the increase and then again *n*/2 qubits for the decrease. If the circuits detect a sharp increase (decrease) before the intended peak (valley) value and then a shard decrease (increase) after the value then the test is successful and the peak (valley) has been found.

We will now see that practical experiments can be conducted on tiny examples only and reveal that quantum computers are yet neither large enough nor reliable enough to be useful in such computations.

## Quantum circuit experiments and reliability

This section is dedicated to actual implementations of the theoretical circuits presented in the previous section. The implementations are done on the quantum platform offered by IBM QX [7]. The experiments were run on a Falcon r4 processor, and most of the experiments had available 5 input qubits and a quantum volume of 16. All experiments have a standard number of 1024 runs.

As the size of the input, the breadth of the circuit, and the volume of the circuit need to be small, the circuits and experiments presented here give a proof of concept, but cannot be applied to large numbers. In fact all operations, such as subtraction, absolute value, etc. are executed on one to three qubit data input only. Additional input qubits are necessary for computations and partial results. The analysis of the results show that even in these small situations, the quantum computer does not offer enough reliability for a usable result.

### One qubit subtraction circuit

The subtraction of two quantum registers is used in all algorithms described in the previous section. Given the size of IBM Qx, the circuits described below are operating one qubit subtractions only. The circuits are meant to be the building block of a subtraction of registers of arbitrary size, in which case they are repeated in series for all qubits in the input registers. Recall, that a one bit subtraction, see Table 1 needs three inputs:

1. $q_0$ is the carry qubit from the previous bit subtraction.
2. $q_1$ is the minuend.
3. $q_2$ is the subtractor qubit.

The subtraction calculates the result and the new carry. We designed two circuits. The first circuit computes the one qubit result, that is the actual difference, shown as the fourth column in the table. The second circuit computes the new carry value to be sent to the next subtraction circuit unit, shown as the fifth column in the table.

**Table 1:** This table shows the expected bit values for a simple one bit subtraction

One Bit Subtraction and Carry				
Carry	Q1	Q2	Result	New Carry
0	0	0	0	0
0	0	1	1	1
0	1	0	1	0
0	1	1	0	0
1	0	0	1	1
1	0	1	0	1
1	1	0	0	0
1	1	1	1	1

Each of the qubits in the circuits can be in superposition. If, let us say, the minuend $q_1$ is a superposition of two states and $q_2$ is a superposition of two states, then the results will be a superposition of the differences of all pairs of components, namely fours differences. In the same way, $q_2$, the carry qubit, is a superposition for all possible subtraction carry values obtainable from the operations on less significant digits. For example, in the algorithm of Quantum circuit for near to constant values section, the minuend is a qubit that holds a classical bit value, as this value is unique to the point for which we study the property, whereas, the subtractor is a superposition of the values of the neighbors. In the algorithm presented in Fig. 8 the opposite is true, namely the subtractor is in superposition and the minuend is a simple state. The fact that the circuit works on superpositions is a powerful tool, as all subtractions are computed using quantum parallelism.

#### The result of the subtraction

Figure 7 shows the circuit that computes the result of the subtraction. The first block shows the input. In the particular case of the figure, the input qubits are set to $q_0=1$, $q_1=1$, and $q_2=0$. The second block of the circuit sets the result according to the expected values as defined in Table 1. The third and last block performs a measurement of the qubits. The only qubit of interest at this stage is qubit $q_3$. This circuit gives the correct result theoretically on all inputs. This has been tested both by following the logic as well as in the experiments on IBM Qx.

**Fig. 6:**
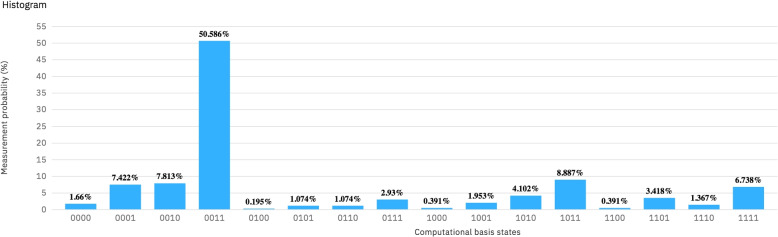
Histogram showing the measurements for the circuit in Fig. 9. The theoretical expected results appears 50.586 % times

Nevertheless, the actual runs of the circuit give very different results. For example, on the input shown in Fig. 1, there are measured results for all possible outcomes, see Fig. 6. The histogram shows a clear peak for the theoretical expected result $|0011\rangle$, but the occurrence of 50% of this result is far from acceptable. Note that the qubit of interest is $q_3$ which in this case is 0. All other qubits may have been destroyed from their original values. If we add all probabilities where $q_3=0$ the success rate is better, namely 72.754 %.

The circuit has been run on all possible basic inputs, namely on eight inputs according to Table 1. The results for all this inputs are collected in Table 2. For each input, the probability of success is shown both as following the theoretical result only, this is column 6, and then the probability of success when only correctness of the result $q_3$ is taken into consideration. We can see that the overall rate of success ranges from 59.083 % to 77.247 %. Consider that errors propagate along the register of size *s*, showing the need for improving the accuracy of the quantum computer.

**Table 2:** This table shows the expected bit values for a simple one bit subtraction. The circuit computes the difference

Measurements for the Result of Difference Circuit						
Q0 - Previous Carry	Q1 - Minuend	Q2 - Subtrahend	Q3 - Difference	Expected Measurement	% of the Expected Measurement	% of Correct Q3
0	0	0	0	$|0100\rangle$	61.426	75.293
0	0	1	1	$|1000\rangle$	66.016	77.247
0	1	0	1	$1010|\rangle$	35.059	72.267
0	1	1	0	$|0110\rangle$	42.871	65.918
1	0	0	1	$|1101\rangle$	48.047	59.083
1	0	1	0	$|0001\rangle$	58.105	70.703
1	1	0	0	$|0011\rangle$	50.586	72.754
1	1	1	1	$|1111\rangle$	50.098	69.728

#### The new carry of a subtraction

**Fig. 7:**
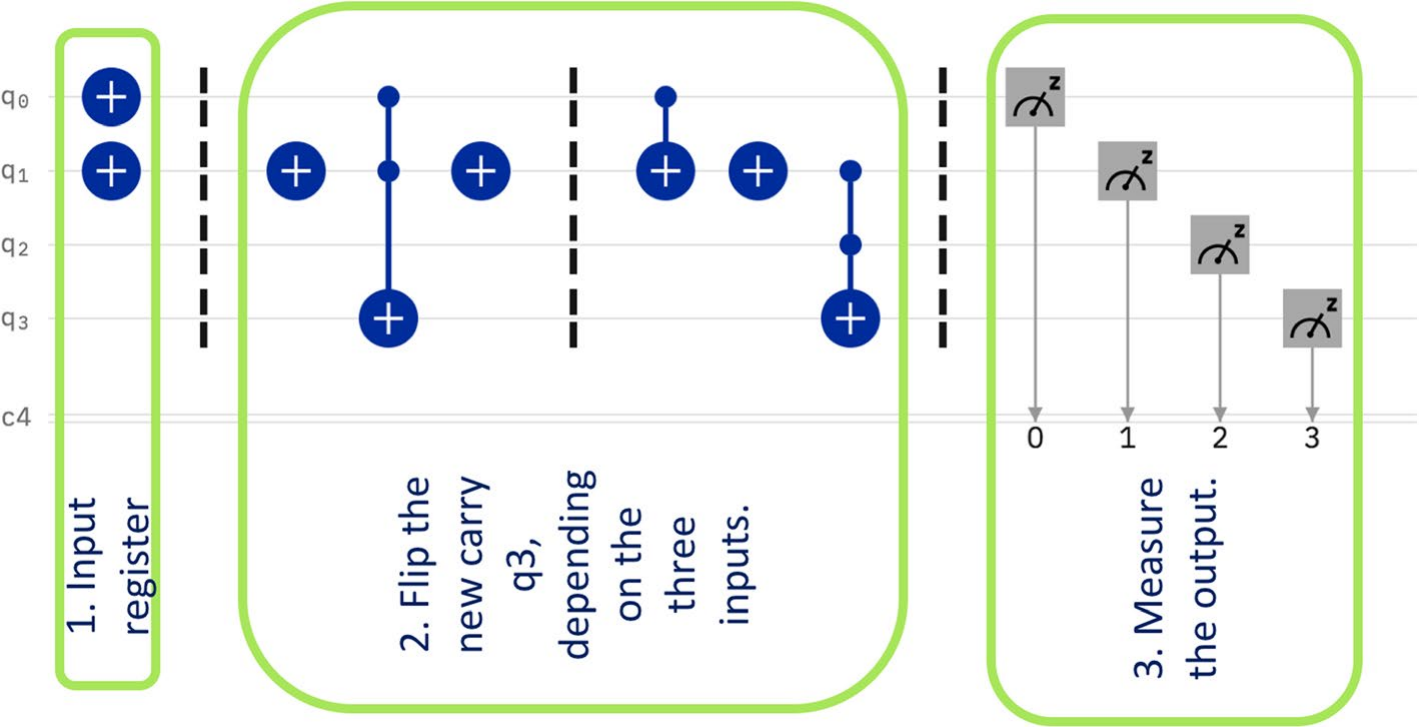
This is part of the circuit that computes the subtraction of two qubits and includes a carry from the previous operation. The last qubit is the new carry

In the second circuit, Fig. 7, the output of the circuit, $q_3$ is the new carry value to be fed to the next significant computation. It has the same three stages as the circuit in the previous section. The second stage, the one that actually computes the new carry. is different in content. It follows the logic of the last column in Table 1.

This circuit, theoretically, always gives the correct answer. In practical runs, the quantum computer again shows a steady percentage of errors. The circuit has been run on all possible inputs and a run on each particular input consists of 1024 actual runs of the quantum circuit. Table 3 shows the percentages for correct measured outcomes. the sixth column shows the percentage of measurement of the theoretical expected value. We see that the lowest value is for an input of $q_0=0$, $q_1=1$, and $q_2=0$, that is 39 %. The last column shows all the measurements where $q_3$ yields the correct values, even if the input qubits were not measured at the expected value. Here, the percentages are between 57 % to 70 %. It shows that in all cases the correct output is more abundant.

**Table 3:** This table shows the expected bit values for a simple one bit subtraction

Measurements for the Carry Circuit						
Q0 - Previous Carry	Q1 - Minuend	Q2 - Subtrahend	Q3 - New Carry	Expected Measurement	% of Expected Measurement	% of Correct Q3
0	0	0	0	$|0010\rangle$	45.605	61.426
0	0	1	1	$|1110\rangle$	53.125	69.921
0	1	0	0	$|0000\rangle$	39.453	70.312
0	1	1	0	$|0100\rangle$	42.676	57.617
1	0	0	1	$|1001\rangle$	54.492	70.899
1	0	1	1	$|1101\rangle$	43.555	67.385
1	1	0	0	$|0011\rangle$	39.844	60.352
1	1	1	1	$|1111\rangle$	40.625	64.453

### Absolute value circuit

Negative numbers are usually recorded in the two’s complement format. Therefore, computing the absolute value of a number means simply to compute the two’s complement of negative values, while leaving positive values unchanged. The description of the two’s complement is standard and can be found in [13]. Our experiment works on a three qubit register and computes the absolute value according to Table 4. The third column shows the absolute value of the three bit number shown in the first column.

**Table 4:** This table shows absolute value computation of a three bit register

Absolute Value of a Three Bit Register			
Q2-Q1-Q0	Q3 - Helper qubit	New Register	% of Correct Measurement
000	0 $\rightarrow$ 0	000	81.835
001	0 $\rightarrow$ 0	001	70.804
010	0 $\rightarrow$ 0	010	70.023
011	0 $\rightarrow$ 0	011	68.261
100	0 $\rightarrow$ 0	000	83.691
101	0 $\rightarrow$ 1	011	67.480
110	0 $\rightarrow$ 0	010	70.019
111	0 $\rightarrow$ 1	001	71.679

**Fig. 8:**
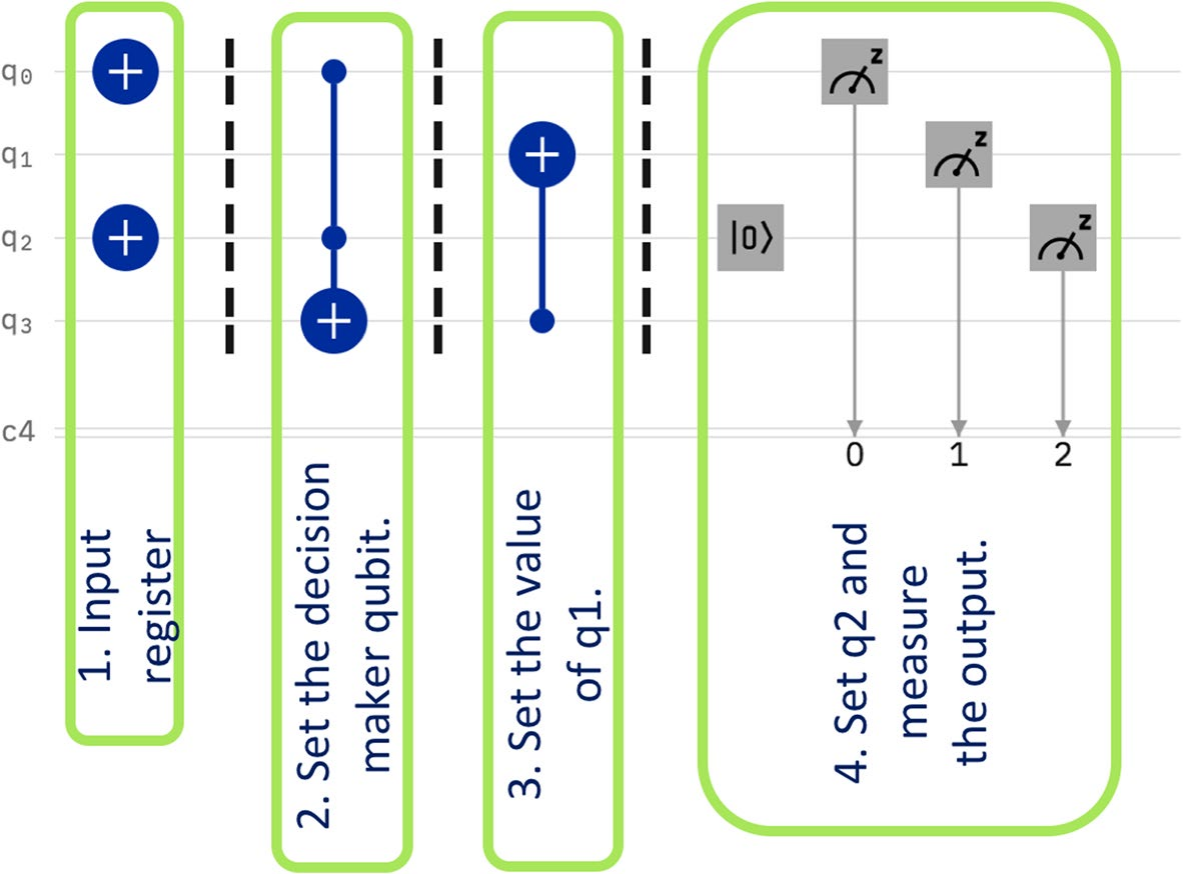
Compute the absolute value of a four qubit register

The quantum circuit that computes the absolute value of a quantum register of 4 qubits is shown in Fig. 8. The input register is fed into the qubits $q_2 q_1 q_0$, where $q_0$ is the least significant digit and $q_1$ is the most significant digit, while $q_2$ represents the sign. Qubit $q_3$ is used as decision maker, it shows whether qubit $q_1$ of the input register needs to be flipped or not.

The circuit has four stages and each stage performs a separate operation. The first stage represents the setting of the input. In Fig. 8 the particular value of the input is 101. In the second stage the decision making qubit $q_3$ is set. In the third stage, qubit $q_1$ gets flipped if necessary. Note that in the computation of the complement of two, the least significant bit is never flipped. Finally, in stage 4, the sign is adjusted to be always positive in the output and then the qubits are measured.

This circuit has also been run on all possible input values, with 1024 times runs for each input. The results are shown in Table 4. The percentages vary between 67% to 83%. This result falls within an approximate similar range to the previous two circuits.

We are ready to evaluate the results for all circuits and to draw conclusions about their applicability.

## Discussion and analysis

**Fig. 9:**
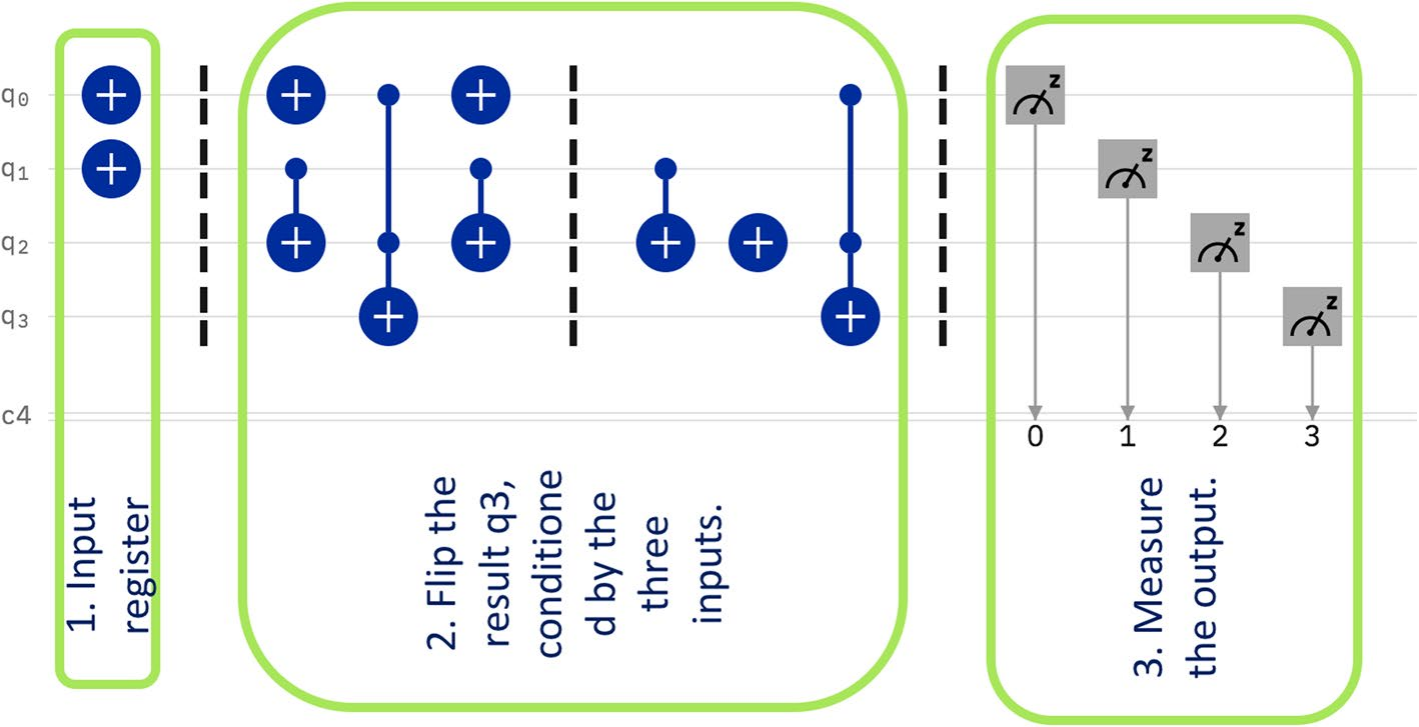
This is part of the circuit that computes the subtraction of two qubits and includes a carry from the previous operation. The last qubit is the result of the subtraction

**Fig. 10:**
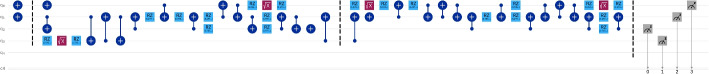
Compute the absolute value of a four qubit register

We have experimented with three circuit implementations: computing the difference, computing the carry of a difference and computing the absolute value. From the figures given for these circuits, we see that the breadth of all these circuits is 4 qubits. The depth of the circuits vary from 4 gates, in Fig. 8 to 7 gates, in Figs. 7 and 9. Note that some gates are controlled NOT gates: simple controlled with one control qubit CNOT, or double controlled with two control qubits CCNOT. When the circuits are run on the quantum computer they are first transpiled (translated) into the physical gates that the computer can perform. CCNOT gates do not exist directly in the quantum hardware and are implemented by simple controlled gates. As an example, we show the transpiled circuit for the absolute value circuit, see Fig. 10. The transpiled circuit has a depth of 40. Table 5 shows all the aggregated results for the three circuits.

**Table 5:** Aggregated reliability data for the practical circuits

Absolute Value of a Three Bit Register					
Name of the Circuit	Worst Accuraacy	Best Accuracy	Breadth	Depth of the Initial Circuit	Depth of the Transpiled Circuit
Difference Result	59 %	77 %	4	7	40
Difference Carry	57 %	70 %	4	7	40
Absolute Value	67 %	81 %	4	4	25

Denote the worst accuracy with *w*. The overall minimum in accuracy in the table is $w=57 \% = 0.57$ and the worst error rate is $e=100\%-57\% = 43 \% = 0.43$. We will consider this as the worst case for further analysis. The worst accuracy has been measured for the difference on one qubit only. For a register of size *s*, the one qubit circuits are serialised and the error accumulates. The accuracy rate for an *s* qubit register becomes $w^s$. The value of *s* is given by the dimensionality of the squiggle values. The raw squiggle data values, as represented in Fig. 2, vary between 200 to 600 mV with an effective step size of 1 mV. This gives a range of 400 values. Thus to represent 400 values, we need the register of size *s* to be able to hold 400 different values: $2^s = 400$ and then $s \approx 9$. Thus, the accuracy at the end of a subtraction with carry computation is approximately $w^s = 0.57^9 = 6.35 * 10^{-3}$. This is way below anything usable. We can conclude that in order for this method to work, an error correction method needs to be used.

We will show methods to increase the accuracy to an arbitrary value, by using

1. Error correcting techniques
2. Reducing the dimensionality of the squiggle data.

Suppose that this error rate has to be improved to a very small number, for example $\epsilon = 0.01$. This can be done by using more qubits to encode one single qubit value and then use an error correction code, such as he Hadamard code [1].

We consider that the alphabet has only two options: zero and one. Therefore, a Hadamard encoding is not justified. We can simply assign *n* qubits to represent one qubit and let the majority measurement decide on the value of the qubit. In this case, $n/2-1$ errors are allowed to get the correct answer. The error rate can be calculated by $$error = \left( {\begin{array}{c}n\\ 0\end{array}}\right) e^n + \left( {\begin{array}{c}n\\ 1\end{array}}\right) e^{n-1} w + ... \left( {\begin{array}{c}n\\ \frac{n}{4}\end{array}}\right) e^{n-\frac{n}{4}} w^{\frac{n}{2}} < \epsilon$$ For example, for $n=16$, we can calculate $error=0.37$. This is a small improvement from 0.43. Thus, we can conclude that the quantum computers to date need to improve their accuracy internally to be useful for this type of circuits.

The size of a quantum computer that can deal with the regular squiggle data with a data space of 400 integer values can be evaluated in the following way. The register size has to be $s=9$ and the circuits presented for one qubits linearly add up. Thus, the breadth of a circuit for regular squiggle values is derived from Table 5$breadth=4*s = 36$, the depth of the circuit needs to be $depth=40*s = 360$ and the volume needs to be $volume=160*s = 1140$ To reduce the necessary size of the quantum computer, the second approach is to work on minimizing the squiggle data dimensionality. We have seen in Squiggles section that the data space can be reduced from 400 integer values to 50 integer values without losing the data property. I this case, $s=6$ and the new quantum computer size needed is: $breadth=24$, $depth=240$, and $volume=960$. These are already quite reasonable values. Note that this is the size as defined for one operation only. The operations along the squiggle data array itself would still be sequentially fed into the quantum computer.

Our analysis reveals that Nanopore squiggle data interpretation through a quantum approach is possible, though at the moment restricted in feasibility with current hardware.

## Conclusion

Quantum computers of reasonable sizes are expected to improve data analysis of problems requiring a large amount of data. We considered a particular niche of data analysis, namely squiggle data analysis as part of the determination of DNA and RNA sequences. We have shown that quantum specific procedures, such as quantum parallelism can be employed to improve (speed up) feature selection in Nanopore data.

We have applied quantum algorithms to extract features of the Nanopore squiggle data. The main idea is to calculate the desired property in parallel on a superposition of consecutive Nanopore current values and extract the result using Grover’s enhancement algorithm. Thus, the complexity of the operation is reduced in theory by a factor of $\sqrt{n}$ for *n* operations performed in parallel. As Nanopore data is considered to be large, this is expected to be a significant improvement.

Additionally, quantum circuits have been implemented to show a proof of concept for the theoretical algorithms. Quantum computers available allow implementations with breadths of 4 qubits and a depth of up to 40 gates. In this situation, it has been shown that in the worst case, the error rate is prohibitively large, namely $47 \%$. This number is actually very close to $50 \%$. Theoretically, using coding techniques, the error rate can be decreased arbitrarily, but this requires a large number of additional qubits. We have shown that a multiplicity of 16 is obtaining a small decrease in the error rate only.

Decreasing the dimensionality of the current value range is a promising approach to reduce the quantum resource needs. We have had experiments with a range reduced from 200 to 5.

In conclusion, quantum computers are promising to deal with Nanopore data theoretically. The size of a quantum computer to be able to deal with such data has to grow with one or two orders of magnitude and offer better reliability.

## Data Availability

Not applicable.
